# Rhinorrhea, cough and fatigue in patients taking sitagliptin

**DOI:** 10.1186/1710-1492-6-8

**Published:** 2010-05-12

**Authors:** James N Baraniuk, Mary J Jamieson

**Affiliations:** 1 Division of Rheumatology, Immunology, and Allergy, Georgetown University, Washington, DC, USA; 2 Department of Family Medicine, Quillen College of Medicine, East Tennessee State University, McMinnville, TN, USA

## Abstract

Sitagliptin is a dipeptidyl peptidase-4 (DPP IV, CD26) inhibitor indicated for treatment of Type II diabetes as a second line therapy after metformin. We report fifteen sitagliptin intolerant patients who developed anterior and posterior rhinorrhea, cough, dyspnea, and fatigue. Symptoms typically developed within 1 to 8 weeks of starting, and resolved within 1 week of stopping the drug. Peak expiratory flow rates increased 34% in 8 patients who stopped sitagliptin. Similar changes were found in 4 out of 5 persons who had confirmatory readministration. Chart review identified 17 patients who tolerated sitagliptin and had no symptomatic changes. The sitagliptin intolerant group had higher rates of clinically diagnosed allergic rhinitis (15/15 vs. 6/18; p = 0.00005), Fisher's Exact test) and angiotensin converting enzyme inhibitor - induced cough (6/13 vs. 1/18; p = 0.012). Nasal and inhaled glucocorticoids may control the underlying allergic inflammation and abrogate this new sitagliptin - induced pharmacological syndrome. Potential mucosal and central nervous system mechanisms include disruption of neuropeptides and/or cytokines that rely on DPP IV for activation or inactivation, and T cell dysfunction.

## Background

Sitagliptin is a selective dipeptidylpeptidase-4 (DPP IV, CD26, EC 3.4.14.5) inhibitor indicated for the treatment of Type II diabetes mellitus [1]. Diabetics treated with sitagliptin (Januvia™, Merck & Co., Inc., Whitehouse Station, N.J.) develop "upper respiratory tract infections", "cough", and "sore throat" in 5% to 6% of subjects [2]. Similar rates for these adverse events have been reported for the other DPP IV inhibitors vidagliptin [3] and saxagliptin [4]. Infections from all causes had a 34% relative risk increase (95% confidence interval 10% to 64%, P = 0.004) for sitagliptin compared to other diabetes treatments [5]. Previous studies have predicted that airway adverse events may occur with this class of drugs [6-9]. We propose that inflammatory changes may be occurring that were coded as infections in clinical studies. This is of importance in balancing the risk: benefit ratio for treatment with DPP IV inhibitors [10,11].

Two subjects who had recently started taking sitagliptin presented to our clinics with rhinorrhea, cough, dyspnea and fatigue, and requested evaluations for drug sensitivity. We challenged these index cases to determine if sitagliptin induced a reproducible syndrome. When the challenges were affirmative, we reviewed charts to identify other sitagliptin - treated subjects. We identified sitagliptin intolerant and tolerant groups, and began an analysis of potential mechanism(s) and risk factors for this new drug - induced syndrome.

## Methods

The index cases were type II diabetic subjects who presented to an urban tertiary allergy center and a rural family practice clinic with upper and/or lower airway symptoms shortly after starting oral sitagliptin (25 and 100 mg per day, respectively). Chart reviews at the rural clinic identified 205 diabetics including 31 who had received sitagliptin as an adjunct to combinations of metformin, sulfonylurea and insulin. Symptoms of fatigue, anterior and posterior rhinorrhea, cough, and sensations of wheezing or dyspnea defined a "sitagliptin intolerant population". Fifteen intolerant and seventeen tolerant patients were identified and examined for potential risk factors and mechanisms of sitagliptin - related complaints. Outpatient evaluations included history, review of medication - related adverse events, physical examination, and, when possible, measurement of peak expiratory flow rates. Spirometry and allergy skin tests were performed at the urban clinic. Peak expiratory flow rate (PEFR) and subjective impressions of anterior and posterior nasal discharge, cough, dyspnea, and fatigue symptoms scores (0 to 10 ordinal scales with 0 = none and 10 = worst in life) were assessed by the physician at the visit when sitagliptin was stopped, and by the patient for a 1 to 2 week follow-up period. Health insurance restrictions and referral opportunities precluded allergy testing for most of rural diabetics. Clinical diagnoses of allergic rhinitis and asthma were inferred from Allergic Rhinitis In Asthma (ARIA) [12] and Global Initiative for Asthma (GINA) [13] guidelines. Specific details are given in the Case Reports.

The diagnosis of allergic rhinitis was made clinically using the symptom algorithm of the ARIA guidelines [12]. These rhinitis subjects had rhinitis with itch, sneezing, watery nasal and ocular discharge that was improved by nasal glucocorticoids, monteluklast, and/or antihistamine therapy during their target season(s). This rural patient population was unique because tree nursery farms were the chief agricultural industry in this naturally forested geographical area. The non-indigenous trees contributed a large additional burden to the high levels of diverse hardwood forest pollens. Community members paid careful attention to the timing of eye and nose itching, sneezing, congestion and cough symptoms in the setting of widespread commercial knowledge of pollination times for each cultivar. Allergic rhinitis was diagnosed frequently (19/31, 61%) in this group. A subsequent analysis of 330 consecutive practice patients found that 59% met allergic rhinitis criteria using the ARIA algorithm [12]. This compares to 42.5% in the 2005-2006 U.S. National Health and Nutrition Examination Survey where atopy was defined by having at least one positive result to 15 allergen tests [14]. Five patients (Cases 1, 3, 6, 7, 21) had positive skin tests to further support their diagnosis.

Five patients wanted to restart the drug. Two wanted to know if sitagliptin was responsible for their symptoms, while three others tried because of its beneficial hypoglycaemic and weight effects. Each patient was counselled about the probable return of symptoms according to clinical standards of care. Patients measured PEFR and clinical symptoms after restarting the sitagliptin to assess drug effects. This amounted to a dechallenge - rechallenge paradigm [15,16]. Placebo, nocebo and other related effects must be considered in reviewing the results of these open drug administrations [17-19].

Statistical differences between groups were determined by two-tailed unpaired Student's t-tests and Fisher's Exact test.

## Results

Thirty three diabetics using sitagliptin were identified. Fifteen intolerant patients had combinations of fatigue, anterior and posterior rhinorrhea, cough, sensations of wheezing, and dyspnea. Four had obesity - related restriction on spirometry. Eighteen patients were tolerant to sitagliptin and did not develop these symptoms.

**Figure 1:**
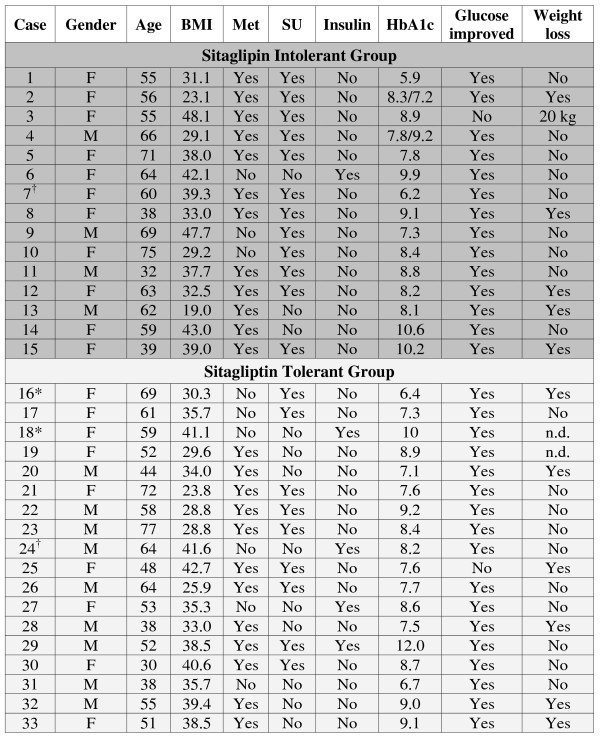
**Demographics, treatments and responses for the sitagliptin intolerant and tolerant groups**. Treatments were metformin (Met), sulfonylureas (SU) and insulin. (* Cases 16 and 18 were treated with chronic low dose methotrexate for rheumatoid arthritis. ^† ^Case 7 died of a pulmonary embolism, and Case 24 had a sudden unexplained death. n.d., not determined.)

Significantly more of the intolerant individuals had allergic rhinitis (15/15) than the sitagliptin tolerant (6/18) group (p = 0.00005). Angiotensin converting enzyme inhibitor (ACEI) intolerance was more common in those intolerant to sitagliptin (6/13) compared to tolerant patients (1/18; p = 0.012). Overall, patients with a clinical history of allergic rhinitis had more ACEI intolerance (7/19) than patients without that history (0/12) (p = 0.019). The two groups were equivalent for age, gender, hemoglobin A1c, the proportions treated with metformin, sulfonylureas and insulin, sitagliptin doses, and rates of improved glucose control and weight loss on sitagliptin (p > 0.20) (Figure 1). These patients were taking multiple medications as is typical for diabetic patients. The most common were ranked as ACE inhibitors, statins, other antihypertensives and antidepressant medications. Their use was similar in both groups. Each patient's combination of medications was evaluated for cytochrome P450 and other drug interactions that could have caused similar patterns of symptoms. However, none were found.

**Figure 2:**
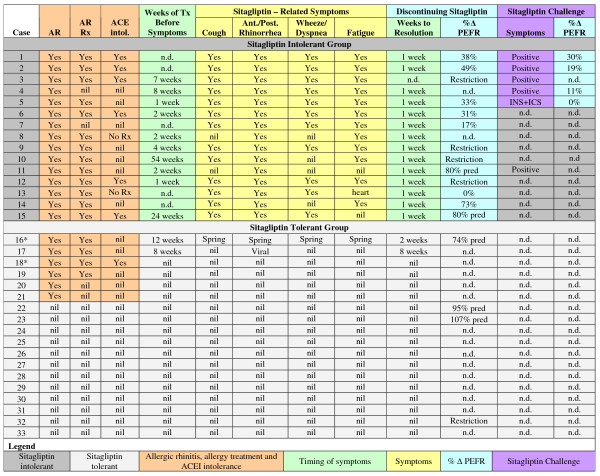
**Sitagliptin adverse events**. The presence of allergic rhinitis (AR) and its treatment (AR Rx), ACE inhibitor intolerance (ACE intol.), duration of sitagliptin treatment before symptoms began, the nature of the symptoms, and effects of discontinuation and subsequent challenge are shown. PEFR values are shown as % change after stopping sitagliptin, or normalized % of predicted (% pred) for tolerant subjects. Five subjects had restrictive patterns on spirometry that may have been related to obesity. Their PEFRs were not reported. Effective use of intranasal and inhaled corticosteroids (INS+ICS) prevented the return of symptoms for Case 5. Cases 16 and 18 used chronic methotrexate for rheumatoid arthritis (*). Case 23 developed a rash with sitagliptin which recurred on rechallenge. Case 9 now requires hemodialysis for hypertensive renal failure. Case 25 developed non Hodgkins lymphoma. Case 7 died from a pulmonary embolism. Case 24 had a sudden unexpected death. (nil, no complaints; n.d., not determined).

All sitagliptin intolerant subjects had seasonal or perennial allergic rhinitis treated with intermittent antihistamines and nasal steroid sprays (Figure 2). Those with mild intermittent asthma generally had been prescribed montelukast, an inhaled glucocorticoid or inhaled albuterol which were used on an *ad hoc *basis. The median time for onset of sitagliptin - related symptoms was 3 weeks (range 1 to 8 weeks) except for Cases 10 and 15. Case 10 began taking sitagliptin during ragweed season while taking montelukast and had no adverse symptoms. However, during the next ragweed season she developed intolerable rhinitis symptoms despite the montelukast. Symptoms resolved within a week of stopping sitagliptin even though the ragweed season continued unabated. Case 15 began having symptoms during the local grass season. Symptoms persisted for months into the winter and resolved within a week of stopping sitagliptin.

Anterior and/or posterior rhinorrhea, fatigue, cough and the sensation of wheezing or dyspnea developed in all eleven intolerant patients, with the following exceptions. Fatigue may have been related to concomitant ischemic heart disease in Cases 6, 12 and 13. Obesity - related airflow restriction was present in Cases 3, 9, 10 and 12. Case 5 had no wheeze or dyspnea, while Case 8 had no cough, wheezing or dyspnea. Case 11 did not have symptoms during a first, short trial with sitagliptin, but developed rhinitis when the drug was restarted during his usual, symptomatic, tree pollen season. His symptoms disappeared within 1 week of stopping sitagliptin.

PEFR increased between 0% and 73% after sitagliptin was stopped. Overall, PEFR increased 34% (23% to 44%) (mean, 95% confidence interval) following cessation of sitagliptin treatment and challenges. However, only Cases 2 and 14 had significant changes in spirometry (e.g. FEV1/FVC) suggesting that reduced PEFR on sitagliptin may have been related to potential decreases in effort without intrapulmonary bronchoconstriction.

Rhinorrhea, cough and fatigue generally improved in the first week off sitagliptin, while PEFR took 1 to 3 weeks to improve. Sitagliptin was readministered to five intolerant patients (see Case Reports). Four had an identical set of symptoms recur showing the reproducibility of their responses (Figure 2). The fifth person had moderate allergic rhinitis with mild seasonal asthma, but became symptom free after becoming highly compliant with intranasal and inhaled mometasone furoate. This suggested that proper identification of atopy and institution of indicated glucocorticoid therapy prevented the adverse airway effects of sitagliptin. This rural population seems to have had underappreciated their mild intermittent asthma, and so were undertreated.

Seventeen patients were tolerant to sitagliptin and did not develop syndromic rhinorrhea, cough, fatigue, dyspnea or sensation of wheezing with the drug. However, two did develop some symptoms. Case 16 developed cough, rhinorrhea, wheeze and fatigue during tree pollen season with PEFR 74% of predicted. Sitagliptin had no effect on the pattern of symptoms. Rhinitis and asthma symptoms resolved within 2 weeks of initiating nasal and inhaled fluticasone propionate. Nasal fluticasone and the use of methotrexate for rheumatoid arthritis prevented recurrence of symptoms during grass and ragweed seasons. Case 16 had viral rhinitis lasting 8 weeks. Montelukast controlled the seasonal rhinitis. Cases 18 and 19 took nasal steroids and did not develop symptoms. Case 18 had long standing rheumatoid arthritis treated with methotrexate. Case 20 developed seasonal rhinitis symptoms which improved with nasal steroids the year after stopping sitagliptin. Case 21 had completed immunotherapy years before sitagliptin administration and did not develop symptoms. The remaining eleven subjects had none of these symptoms. Two had normal spirometry and one had obesity - related restriction.

## Case Reports

### Case 1

**Figure 3:**
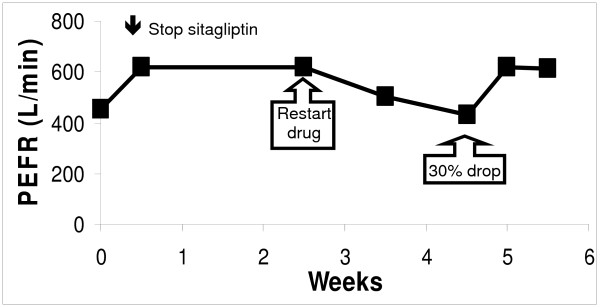
**Case 1**. Increased dyspnea was noted while on sitagliptin (week 0). Peak flows increased rapidly within several days of stopping the drug. Restarting sitagliptin after 2 1/2 weeks led to a progressive, 30% decline in PEFR. PEFR again returned to her usual after stopping the drug (weeks 5 and 6).

A 55 yr old, atopic, white female developed Type II diabetes. She had hypothyroidism, ragweed-induced seasonal asthma, hypertension and history of ACEI cough. She started sitagliptin 100 mg by mouth daily in the early winter and then developed nasal congestion, post-nasal drip, and a throat-clearing cough. A frontal headache developed that gradually worsened over time. She decided to stop the drug when her peak expiratory flow rate (PEFR) dropped to 450 L/min (Figure 3). The next day her headache and congestion were gone. The cough ceased 3 days later. PEFR rose to 620 L/min. She also noticed more vigor and realized she had become very fatigued on sitagliptin. She requested a supervised course of sitagliptin (50 mg) to determine if these symptoms represented a reproducible, drug - induced syndrome. Symptoms recurred over the next 3 days. Her lowest PEFR was 430 L/min after 2 weeks. She scored congestion severity, post-nasal drip, throat clearing and tiredness/decreased energy at 5 to 8 out of 10 and headache as 5 to 7 out of 10. Cough was intermittent during these 2 weeks. After stopping the drug, all symptoms disappeared and PEFR returned to her normal.

### Case 2

A 55 year old, white female had Type II diabetes, hypothyroidism, hypertension with history of ACEI cough, persistent mild allergic rhinitis with seasonal worsening to moderate levels, and chronic moderate persistent asthma. After starting sitagliptin 100 mg by mouth daily, she developed severe rhinorrhea and cough which persisted for months.

When she returned for follow-up, her PEFR was 176 L/min. Her FEV1/FVC was 63% and FEF25%-75% was 43% of predicted. Sitagliptin was stopped. She scored her postnasal drip as 3/10 two days after stopping the drug. The cough resolved over several days and her PEFR rose to 280 L/min after 12 days.

Later, she asked to restart sitagliptin because of the beneficial hypoglycaemic benefits. Unfortunately, this was during her typical tree pollen-induced rhinitis period. PEFR dropped to 180 L/min and rhinorrhea scores increased to 9/10 despite the concomitant, but intermittent, use of inhaled steroids and bronchodilators. Cough did not recur, but she stopped the sitagliptin because of dyspnea. Dyspnea resolved within one day, and rhinorrhea in 2 days (0/10). Two weeks later, her FEV1/FVC had increased to 79.1%, FEF25%-75% to 69% of predicted, and PEFR to 320 L/min. The next year while off sitagliptin, her maximum rhinorrhea score during the tree pollen season was 5/10.

Fatigue at the end of her first sitagliptin treatment period was 7/10. This dropped to 4/10 after stopping the drug. During the challenge period, her fatigue again reached 7/10. Fatigue decreased to 3/10 within 3 days of stopping the sitagliptin challenge, and remained low throughout her tree pollen rhinitis season.

### Case 3

A morbidly obese 55 yr old African-American woman had metabolic syndrome, atopic and aspirin-induced asthma and rhinitis, and history of ACEI cough. Atopy and asthma were controlled by inhaling one puff of 500 μg fluticasone propionate/50 μg salmeterol twice per day, fexofenadine (180 mg), nasal mometasone, occassional nebulized budesonide (0.25 mg) plus levalbuterol treatments, and omalizumab (375 mg sc every 2 weeks). Sitagliptin (50 mg per day) was added to metformin and glucophage. She noted progressive fatigue and loss of energy, but no cough or alteration in her intermittent pattern of wheezing. Although she lost 20 kg, sitagliptin did not improve glucose control, and so it was discontinued. Several months later the drug was restarted to maintain the weight reduction. Eight weeks later she reported increased fatigue (4/10 became 8/10), cough (0/10 rose to ?6/10), and dyspnea (5/10 increased to 8-9/10). PEFR was persistently low at 250 L/min. She promptly developed a parainfluenza infection complicated by acute rhinosinusitis that required azithromycin, and a prolonged asthma exacerbation that required 6 weeks of prednisone and nebulized budesonide (0.5 mg) and levalbuterol (four times per day). This was her worst exacerbation in over three years, and was temporally related to restarting the sitagliptin.

### Case 4

This 66 yr old male developed fatigue, rhinorrhea, cough and sensation of wheezing after 8 weeks of sitagliptin. These symptoms cleared after discontinuing the drug. Sitagliptin was restarted to determine the relationship to his symptoms. Symptom scores increased to 3/10, but PEFR was not recorded. Again the sitagliptin was stopped. Symptom scores dropped to 1/10 and PEFR improved by 11% after 1 week off sitagliptin. His challenge was for proof of principle and sitagliptin was discontinued before severe symptoms developed.

### Case 5

Sitagliptin caused rhinorrhea, cough, dyspnea and fatigue in this 71 yr old female. Symptoms cleared after stopping the drug. She had moderately severe allergic rhinitis with intermittent asthma, but used nasal fluticasone propionate occasionally for relief of the most severe symptoms. However, during sitagliptin challenge, she adhered strictly to daily inhaled and intranasal mometasone furoate. Her symptoms did not recur despite entering her generally severe fall ragweed season. This may suggest that appropriate, prophylactic glucocorticoid treatment of allergic inflammation may prevent the sitagliptin - induced symptom complex.

### Internet Case

An additional subject was identified by an internet search [20]. Cough was the predominant symptom. The subject had a history of ACE inhibitor cough, but had abstained from ACE inhibitors for several months during sitagliptin therapy.

## Discussion

Factors accounting for sitagliptin pathophysiology can be inferred from a review of DPP IV function and precedents set by other peptidases. This is highly relevant for allergists who may see patients with similar symptoms or apparent drug reactions. The most recent NICE clinical guidelines recommend addition of a DDP IV inhibitor as second line treatment with metformin instead of a sulphonylurea to avoid hypoglycemia [21]. Therefore, physicians may prescribe sitagliptin more often for diabetes control. The mechanisms of DPP IV in vivo may also be relevant to recent reports of 88 cases of pancreatitis by the FDA [22]. We did not encounter any pancreatitis in our study cohort.

DPP IV is a 110 kDa cell surface glycoprotein with serine exopeptidase activity that cleaves proline dipeptides from the N-terminus of polypeptides. In diabetes it cleaves the N-terminal tyrosine-proline dipeptide from the glucagon-like peptide-1 (GLP-1), glucose-dependent insulinotropic polypeptide (GIP), gastric inhibitory peptide, and pituitary adenylate cyclase activating peptide (PACAP). These incretins are released postprandially from the gut and stimulate insulin secretion [1]. DPP IV promotes hyperglycemia by rapidly inactivating these peptides. However, inhibition of DPP IV maintains the incretins at physiological levels that can increase insulin secretion.

DPP IV is the prototype of the DPP IV activity and/or structure homologue (DASH) protein family [23]. The family includes DPP7, DPP8, DPP9, DPP-IV-β, fibroblast activation protein (FAP), and attractin. The common feature is their specificity for cleaving proline from the N- terminal of proteins and peptides. Many of the activities of DPP IV discussed below were identified using relatively nonselective enzyme antagonists. More specific DPP IV and DPP 8/9 antagonists now suggest that some "DPP IV" actions may be mediated by DPP8, DPP9 or other members of this family.

These effects of DPP IV inhibition on airway and other organ symptoms were predictable given the relationships between ACEI and cough, neutral endopeptidase (NEP, CD10; EC 3.24.11) with neurogenic inflammation, and complement C1 esterase inhibitor and hereditary angioneurotic edema [24,25].

Angiotensin converting enzyme inhibitors (ACEI) are the precedent for respiratory adverse events related to peptidolytic drugs [16]. The mechanism of ACE inhibitor-induced cough remains unresolved, but likely involves the protussive mediators bradykinin and substance P, agents that are degraded by ACE and therefore accumulate in the upper respiratory tract or lung when the enzyme is inhibited [26]. Prostaglandins are stimulated by bradykinin and may contribute to the cough. These mediators likely stimulate Type C vagal afferent neurons to provoke the brainstem cough reflex. The prototypical ACEI, captopril, did not enhance the direct vasodilatory or secretory effects of topically applied vasoactive intestinal polypeptide (VIP), substance P (SP) or calcitonin gene-related peptide (CGRP) in the nasal mucosa of 12 healthy volunteers[27]. This is probably because ACE has a predominant plasma origin in nasal mucosa [28]. ACEI also enhance the function of vasodilatory bradykinin B1 and B2 receptors [29]. ACEI treatment of rats with genetic deficiency of DPP IV leads to tachykinin - mediated (substance P, neurokinin A) peritracheal edema [30]. Bradykinin is less likely to be involved since it is not a DPP IV substrate in rat inflammation [31].

DPP IV can cleave substrates such as eotaxin, regulated on activation normal T cell expressed and secreted (RANTES, CCL5), neuropeptide Y (NPY), substance P, chromogranin B - derived peptides, and other airway peptides [32,33]. Sitagliptin's inhibition of DPP IV activity may disrupt the normal functions of these polypeptides, particularly in inflamed mucosa [6].

NEP degrades, and so regulates, the duration of action of many small neuropeptides [34]. Like DPP IV, NEP has reduced expression in chronic rhinosinusitis [7]. This may reduce mucosal destruction of calcitonin gene related peptide (CGRP) leading to increased nasal venous sinusoid engorgement and mucosal thickening, and enhance neurogenic axon responses ("neurogenic inflammation"). Substance P - induced vasodilation was augmented by DPP IV inhibition in an *in vivo *porcine nasal model [6,8]. This potentially neurogenic effect may be tested in human nasal mucosa using hypertonic saline nasal provocations [35]. Intranasal steroid treatment increases NEP, and potentially DPP IV, expression. These enzymes may be biomarkers of recent mucosal injury and subsequent recovery.

Neuropeptide Y (NPY1-36) is released with norepinephrine from sympathetic neurons. NPY1-36 is an agonist of Y1 receptors on arterioles and arteriovenous anastamoses that cause slow onset, prolonged vasoconstriction and resulting in improved nasal patency [36]. DPP IV removes the N-terminal Tyr-Pro dipeptide from NPY1-36 to generate NPY3-36 [37]. NPY3-36 binds to Y2 receptors that have relative antagonist properties to Y1 receptor activation. Y2 inhibitory autoreceptors on sympathetic nerves halt the release of norepinephrine and co-localized NPY. These autoreceptors are also present on parasympathetic nerves and reduce the release of acetylcholine. Any decrease in the peptidolytic generation of NPY3-36 would decrease the activity of Y2 inhibitory autoreceptors and so augment sympathetic and parasympathetic neurotransmitter release. The clinical consequences are difficult to predict.

Elevated parasympathetic acetylcholine release is probably of clinical relevance given the prevalence of rhinorrhea in our subjects. Cholinergic stimulation of M3 muscarinic receptors on submucosal glands leads to copious glandular secretion [38]. This may generate the rhinorrhea reported by our subjects and the 5.2% of sitagliptin users with nasopharyngitis (placebo = 3.3%) and "upper respiratory tract infection" with the combination of sitagliptin and pioglitazone (6.3% vs. 3.4% in placebo) [2]. Cholinergic hypersecretion may be identified by relief of rhinorrhea when sitagliptin sensitive subjects use an anticholinergic nasal spray. Analysis of the nasal secretions may distinguish glandular from vascular sources of the discharge, and the nature of the offending peptide(s). These putative peptide DPP IV substrates may be targets for development of novel rhinorrhea, antitussive, and bronchodilator drugs. Sitagliptin joins the list of drugs associated with nonallergic mechanisms of rhinitis [39].

DPP IV immunoreactive material has been localized to human nasal [6,8] and bronchial [9] mucosa. Immunoreactive material was present in apical (probably serous) cells of submucosal glands, leukocytes, and endothelial cells. Biopsies of human nasal tissue from chronic rhinosinusitis and bronchi in chronic obstructive diseases demonstrated a positive correlation between DPP IV enzyme activity and immunoreactivity. DPP IV enzyme activities in human airway biopsies were inversely related to mucosal inflammatory cell density [6,8,9]. The leukocytes were predominantly memory T cells and monocytes [40-42]. The inverse relationship suggested that products of the inflammatory process inhibited DPP IV expression.

DPP IV is also known as CD26, and is highly expressed on memory T cells [43]. CD26 plays an important role in the proliferation of memory T cells in response to antigen presentation. Activation of CD26 may increase CD86 expression on CD14 positive monocytes and other antigen presenting cells [44].

DPP IV degrades interferon (IFN) gamma - induced chemokines, CCL3, CCL5 (RANTES), CCL11, CCL22, and CXCL12 (stromal cell-derived factor-1 alpha; SDF-1α). This effect may bias mucosal immune responses towards TH2 compared to TH1 lymphocyte phenotype. DPP IV cleavage of the N-terminal dipeptide from CCL5 enhanced chemotaxis of T cells, but not monocytes, in vitro [41].

A soluble form of DPP IV (sCD26) is elevated in asthma. Plasma sCD26 was positively correlated with aberrant expression of cell surface CD26 on a wide range of lymphocytes, altered peripheral eosinophils, Th2-related chemokines CCL5 and CCL22, and the costimulatory molecule soluble cytotoxic T lymphocyte antigen 4 (sCTLA-4) (all P < 0.05) [44]. These cellular mechanisms may augment the consequences of DPP IV inhibitors during tissue inflammation.

Increased attachment of sialic acid residues to the N-linked polysaccharides of DPP IV makes the enzyme more acidic. This may reduce enzyme activity and obstruct access to immunoreactive epitopes and so reduce immunohistochemical staining and immunoassay concentrations. Hypersialylated DPP IV has been recognized in rheumatoid arthritis and systemic lupus erythematosus [45]. Lower activities of plasma sCD26/DPP IV in lupus were correlated with increased disease activity [42]. The addition of sitagliptin under these circumstances of reduced DPP IV activity would further inhibit DPP IV's peptidolytic function.

The novel observation of reproducible, sitagliptin - induced fatigue implicates DPP IV substrates in this neural symptom complex. Fatigue has been associated with ACEI, angiotensin receptor antagonists, direct renin inhibitors, beta blockers, calcium channel blockers, and diuretics [46,47]. However, the relative contributions of underlying hypertension, congestive heart failure, renal disease or diabetes versus altered polypeptide cleavage to fatigue remain to be defined. The reversible fatigue reported by our patients was directly related to stopping and restarting sitagliptin. Identification of the as yet unknown, responsible neurotransmitter(s) or neurotropin(s) offers the potential to understand the molecular pathogenesis of this complex emotional state, and to develop drugs that target these putative mechanisms. Understanding conscious control of cough may also provide insights into the nature of fatigue given their association in this sitagliptin -related syndrome [48].

Central actions of DPP IV inhibitors may be related to weight loss in Type II diabetics. Low plasma sCD26 levels were found in anorexia and bulimia nervosa, [49]. NPY is known to play an important role in hypothalamic control of appetite and satiety. Sitagliptin inhibition of DPP IV may prolong the duration of action of prolyl - peptide substrates that mediate fatigue and weight loss.

Most DPP IV inhibitors have been synthesized with a fluorovinyl (C = CHF) group where the fluorine atom acts like the carbonyl oxygen of a peptide bond [50,51]. However, sitagliptin has triflurophenyl and trifluoromethyl groups that may interact more strongly with amino acid sidechains in the DPP IV active site or other inhibitory locations. Modifications of these groups may lead to more selective DPP IV inhibitors that do not have effects on DPP8, DPP9 or other related peptidases. The more recently released DDP IV inhibitor, saxagliptin, does not have a fluorinated side chain and the "upper respiratory symptom" rate is similar in the treated and placebo groups (7.7%, 7.6%) [4].

Airway inflammation increases mucosal leukocyte density and may decrease glandular DPP IV activity. If so, some DPP IV substrates may have a prolonged half-life as glandular secretagogues. We propose that sitagliptin - induced inhibition of DPP IV activity may supplement this inflammatory effect and lead to augmented peptide - mediated glandular secretion and subsequent post - nasal drip, irritant - induced throat clearing cough, and decreased PEFR. Such a result would be consistent with the clinically defined allergic rhinitis subset of diabetics who also have a tendency for similar ACEI intolerance.

Topical nasal and bronchial glucocorticoids treatments control allergic airway inflammation and may permit DPP IV activity to return to normal. If confirmed, anti-inflammatory treatment may be beneficial for allergic and ACEI intolerant diabetics so that they may continue to safely use sitagliptin when it is clinically indicated for diabetes control.

The allergic patients were identified clinically using the ARIA symptom algorithm. The lack of positive skin testing to confirm allergies is a weakness of this case series. However, diabetic patients presenting to primary care or endocrine clinicians may not have had this testing due to lesser severity of their symptoms, or limitations due to rural location and health insurance coverage of services. Lack of access to spirometry limited the diagnosis of asthma. The unblinded sitagliptin challenges were a logical starting place for determining if the drug was related to the rhinitis, cough and fatigue syndrome. However, placebo and perceptional effects during drug withdrawal may have led to a misattribution of cause and effect. Blinded studies will be needed to confirm our explanation of DPP IV drug adverse events in airways and for fatigue.

## Conclusions

A subset of clinically defined allergic rhinitis subjects had worsening of their symptoms plus fatigue when given sitagliptin. This and other DPP IV inhibitors have been reported to cause "upper respiratory infections" in about 5% of Type II diabetics. We propose that underlying inflammatory changes in DPP IV activity combined with further drug - mediated DPP IV inhibition leads to decreased inactivation of neuropeptides and/or cytokines that are glandular secretagogues. This plus similar mechanism(s) in the brain may be responsible for the rhinorrhea, cough and fatigue we associated with sitagliptin treatment.

## Abbreviations

ACEI: angiotensin converting enzyme inhibitors; BMI: body mass index; CD26; COPD: chronic obstructive pulmonary disease; DPP IV: dipeptidyl peptidase IV; NEP: neutral endopeptidase; NPY: neuropeptide Y; PEFR: peak expiratory flow rate.

## Competing interests

The authors declare that they have no competing interests.

## Authors' contributions

The authors contributed equally to all stages of this project from patient identification and clinical reviews, data analysis, manuscript preparation and editing of the finalized paper.

## References

[B1] Mest HJ Mentlein R Dipeptidyl peptidase inhibitors as new drugs for the treatment of type 2 diabetes Diabetologia 2005 48 616 620 10.1007/s00125-005-1707-5 15770466

[B2] Anon Januvia (sitagliptin) tablets Circular Number 9762802 Merck & Co., Inc. Whitehouse Station, NJ 088893

[B3] Göke B Hershon K Kerr D Calle Pascual A Schweizer A Foley J Shao Q Dejager S Efficacy and safety of vildagliptin monotherapy during 2-year treatment of drug-naïve patients with type 2 diabetes: comparison with metformin Horm Metab Res 2008 40 12 892 5 10.1055/s-0028-1082334 18726829

[B4] Anon Onglyza (saxagliptin) tablets Circular Number 1256316 2009 Bristol-Meyers Squibb, Princeton, NJ 08543

[B5] Richter B Bandeira-Echtler E Bergerhoff K Lerch C Emerging role of dipeptidyl peptidase-4 inhibitors in the management of type 2 diabetes Vasc Health Risk Manag 2008 4 4 753 68 19065993 10.2147/vhrm.s1707 PMC2597770

[B6] Grouzmann E Monod M Landis B Wilk S Brakch N Nicoucar K Giger R Malis D Szalay-Quinodoz I Cavadas C Morel DR Lacroix JS Loss of dipeptidylpeptidase IV activity in chronic rhinosinusitis contributes to the neurogenic inflammation induced by substance P in the nasal mucosa FASEB J 2002 16 1132 1134 12039843 10.1096/fj.01-0939fje

[B7] Lacroix JS Kurt AM Pochon N Bretton C Lundberg JM Deshusses J Neutral endopeptidase activity and concentration of sensory neuropeptide in the human nasal mucosa Eur Arch Otorhinolaryngol 1995 252 465 468 10.1007/BF02114752 8719587

[B8] Giger R Nicoucar K Kurt AM Grouzman E Lacroix JS Study of the enzyme peptidyl peptidase IV in nasal mucosa Schweiz Med Wochenschr Supple 2000 125 99S 101S 11141955

[B9] Landis BN Grouzmann E Monod M Busso N Petak F Spiliopoulos A Robert JH Szalay-Quinodoz I Morel DR Lacroix JS Implication of dipeptidylpeptidase IV activity in human bronchial inflammation and in bronchoconstriction evaluated in anesthetized rabbits Respiration 2008 75 89 97 10.1159/000106267 17637510

[B10] Ahren B Clinical results of treating type 2 diabetic patients with sitagliptin, vildagliptin or saxagliptin--diabetes control and potential adverse events Best Pract Res Clin Endocrinol Metab 2009 23 4 487 98 10.1016/j.beem.2009.03.003 19748066

[B11] Richter B Bandeira-Echtler E Bergerhoff K Lerch CL Dipeptidyl peptidase-4 (DPP-4) inhibitors for type 2 diabetes mellitus Cochrane Database Syst Rev 2008 16 2 CD006739 10.1002/14651858.CD006739.pub2 PMC8985075 18425967

[B12] Costa DJ Bousquet PJ Ryan D Price D Demoly P Brozek J Schunemann HJ Bousquet J Guidelines for allergic rhinitis need to be used in primary care Prim Care Resp J 2009 18 4 250 7 10.4104/pcrj.2009.00028 PMC6619361 19513495

[B13] Bateman ED Hurd SS Barnes PJ Bousquet J Drazen JM Fitzgerald M Gibson P Ohta K O'Byrne P Pedersen SE Pizzichini E Sullivan SD Wenzel SE Zar HJ Global strategy for asthma management and prevention: GINA executive summary Eur Respir J 2008 31 1 143 178 10.1183/09031936.00138707 18166595

[B14] Gergen PJ Arbes SJ Jr Calatroni A Mitchell HE Zeldin DC Total IgE levels and asthma prevalence in the US population: Results from the National Heath and Nutrition Survey 2005-2006 J Allergy Clin Immunol 2009 124 3 447 53 10.1016/j.jaci.2009.06.011 19647861 PMC2758573

[B15] Tumanan-Mendoza BA Dans AL Villacin LL Mendoza VL Rellama-Black S Bartolome M Ragual J Flor B Valdez J Dechallenge and rechallenge method showed different incidences of cough among four ACE-Is J Clin Epidemiol 2007 60 6 547 53 10.1016/j.jclinepi.2006.06.017 17493508

[B16] Israili ZH Hall WD Cough and angioneurotic edema associated with angiotensin-converting enzyme inhibitor therapy. A review of the literature and pathophysiology Annals of Internal Medicine 1992 117 3 234 242 1616218 10.7326/0003-4819-117-3-234

[B17] Eccles R The power of the placebo Curr Allergy Asthma Rep 2007 7 2 100 4 10.1007/s11882-007-0006-2 17437679

[B18] Eccles R Mechanisms of the placebo effect of sweet cough syrups Respir Physiol Neurobiol 2006 152 3 340 8 10.1016/j.resp.2005.10.004 16326149

[B19] Baraniuk JN The placebo effect: plugging the nostrils of unmet needs Curr Allergy Asthma Rep 2009 9 2 149 52 10.1007/s11882-009-0022-5 19210905 PMC4209302

[B20] Januvia and chronic cough - Medications.com http://www.medications.com/se/januvia/chronic-cough

[B21] Adler AI Shaw EJ Stokes T Ruiz F Newer agents for blood glucose control in type 2 diabetes: summary of NICE guidance BMJ 2009 338 1328 9 10.1136/bmj.b1668 19465464

[B22] Information for Healthcare Professionals - Acute pancreatitis and sitagliptin (marketed as Januvia and Janumet) http://www.fda.gov/Drugs/DrugSafety/PostmarketDrugSafetyInformationforPatientsandProviders/DrugSafetyInformationforHeathcareProfessionals/ucm183764.htm

[B23] Veken P Van der Haemers A Augustyns K Prolyl peptidases related to dipeptidyl peptidase IV: potential of specific inhibitors in drug discovery Curr Top Med Chem 2007 7 621 635 10.2174/156802607780091046 17352682

[B24] Skidgel RA Erdös EG Angiotensin converting enzyme (ACE) and neprilysin hydrolyze neuropeptides: a brief history, the beginning and follow-ups to early studies Peptides 2004 25 521 525 10.1016/j.peptides.2003.12.010 15134871

[B25] Frank MM 8. Hereditary angioedema J Allergy Clin Immunol 2008 121 2 Suppl S398 S401 10.1016/j.jaci.2007.07.057 18241690

[B26] Dicpinigaitis PV Angiotensin-converting enzyme inhibitor-induced cough: ACCP evidence-based clinical practice guidelines Chest 2006 129 1 Suppl 169S 173S 10.1378/chest.129.1_suppl.169S 16428706

[B27] Lacroix JS Functional effects of phosphoramidon and captopril on exogenous neuropeptides in human nasal mucosa Eur Arch Otorhinolaryngol 1995 252 2 83 5 7541210 10.1007/BF00168025

[B28] Ohkubo K Lee CH Baraniuk JN Merida M Hausfeld JN Kaliner MA Angiotensin- converting enzyme in the human nasal mucosa Am J Respir Cell Mol Biol 1994 11 2 173 80 8049077 10.1165/ajrcmb.11.2.8049077

[B29] Erdös EG Tan F Skidgel RA Angiotensin I-converting enzyme inhibitors are allosteric enhancers of kinin B1 and B2 receptor function Hypertension 2010 55 2 214 20 10.1161/HYPERTENSIONAHA.109.144600 20065150 PMC2814794

[B30] Byrd JB Shreevatsa A Putlur P Foretia D McAlexander L Sinha T Does MD Brown NJ Dipeptidyl peptidase IV deficiency increases susceptibility to angiotensin-converting enzyme inhibitor-induced peritracheal edema J Allergy Clin Immunol 2007 120 403 408 10.1016/j.jaci.2007.04.012 17531305

[B31] Damas J Bourdon V Liégeois JF Simmons WH Influence of several peptidase inhibitors on the pro-inflammatory effects of substance P, capsaicin and collagenase Naunyn Schmiedebergs Arch Pharmacol 1996 354 662 669 8938667 10.1007/BF00170843

[B32] Ajami K Abbott CA Obradovic M Gysbers V Kähne T McCaughan GW Gorrell MD Structural requirements for catalysis, expression, and dimerization in the CD26/DPIV gene family Biochemistry 2003 42 694 701 10.1021/bi026846s 12534281

[B33] Depreitere J Durinx C Wang Z Coen E Lambeir AM Scharpé S De Potter W Nouwen EJ Presence and release of SR-17 (chromogranin B (586-602)) in the porcine splenic nerve and its enzymatic degradation by CD26/dipeptidyl peptidase IV Regul Pept 2002 106 71 79 10.1016/S0167-0115(02)00038-1 12047913

[B34] Ohkubo K Baraniuk JN Hohman RJ Kaulbach HC Hausfeld JN Merida M Kaliner MA Human nasal mucosal neutral endopeptidase (NEP): location, quantitation, and secretion Am J Respir Cell Mol Biol 1993 9 557 567 8217197 10.1165/ajrcmb/9.5.557

[B35] Baraniuk JN Petrie KN Le U Tai C-F Park Y-J Yuta A Ali M VandenBussche CJ Nelson B Neuropathology in rhinosinusitis Am J Respir Crit Care Med 2005 171 5 11 10.1164/rccm.200403-357OC 15477496

[B36] Baraniuk JN Silver PB Kaliner MA Barnes PJ Neuropeptide Y is a vasoconstrictor in human nasal mucosa J Appl Physiol 1992 73 1867 1872 1282125 10.1152/jappl.1992.73.5.1867

[B37] Kitlinska J Kuo L Abe K Pons J Yu M Li L Tilan J Toretsky J Zukowska Z Role of neuropeptide Y and dipeptidyl peptidase IV in regulation of Ewing's sarcoma growth Adv Exp Med Biol 2006 575 223 229 full_text 16700526 10.1007/0-387-32824-6_24

[B38] Mullol J Baraniuk JN Logun C Mérida M Hausfeld J Shelhamer JH Kaliner MA M1 and M3 muscarinic antagonists inhibit human nasal glandular secretion in vitro J Appl Physiol 1992 73 2069 2073 1474087 10.1152/jappl.1992.73.5.2069

[B39] Staevska MT Baraniuk JN Differential diagnosis of persistent nonallergic rhinitis and rhinosinusitis syndromes Clin Allergy Immunol 2007 19 35 53 10.1027/0838-1925.19.1.35 17153006

[B40] Ohnuma K Munakata Y Ishii T Iwata S Kobayashi S Hosono O Kawasaki H Dang NH Morimoto C Soluble CD26/dipeptidyl peptidase IV induces T cell proliferation through CD86 up-regulation on APCs J Immunol 2001 167 6745 6755 11739489 10.4049/jimmunol.167.12.6745

[B41] Iwata S Yamaguchi N Munakata Y Ikushima H Lee JF Hosono O Schlossman SF Morimoto C CD26/dipeptidyl peptidase IV differentially regulates the chemotaxis of T cells and monocytes toward RANTES: possible mechanism for the switch from innate to acquired immune response Int Immunol 1999 11 417 426 10.1093/intimm/11.3.417 10221653

[B42] Kobayashi H Hosono O Mimori T Kawasaki H Dang NH Tanaka H Morimoto C Reduction of serum soluble CD26/dipeptidyl peptidase IV enzyme activity and its correlation with disease activity in systemic lupus erythematosus J Rheumatol 2002 29 1858 1866 12233879

[B43] Reinhold D Kähne T Steinbrecher A Wrenger S Neubert K Ansorge S Brocke S The role of dipeptidyl peptidase IV (DP IV) enzymatic activity in T cell activation and autoimmunity Biol Chem 2002 383 1133 1138 10.1515/BC.2002.123 12437097

[B44] Lun SW Wong CK Ko FW Hui DS Lam CW Increased expression of plasma and CD4+ T lymphocyte costimulatory molecule CD26 in adult patients with allergic asthma J Clin Immunol 2007 27 430 437 10.1007/s10875-007-9093-z 17525828

[B45] Cuchacovich M Gatica H Pizzo SV Gonzalez-Gronow M Characterization of human serum dipeptidyl peptidase IV (CD26) and analysis of its autoantibodies in patients with rheumatoid arthritis and other autoimmune diseases Clin Exp Rheumatol 2001 19 673 680 11791639

[B46] Chen K Chiou CF Plauschinat CA Frech F Harper A Dubois R Patient satisfaction with antihypertensive therapy J Hum Hypertens 2005 19 793 799 10.1038/sj.jhh.1001899 15951740

[B47] Lam S Choy M Aliskiren: an oral renin inhibitor for the treatment of hypertension Cardiol Rev 2007 15 316 323 10.1097/CRD.0b013e31814852a4 18090068

[B48] Eccles R Central mechanisms IV: conscious control of cough and the placebo effect Handb Exp Pharmacol 2009 187 241 62 full_text 18825344 10.1007/978-3-540-79842-2_12

[B49] van West D Monteleone P Di Lieto A De Meester I Durinx C Scharpe S Lin A Maj M Maes M Lowered serum dipeptidyl peptidase IV activity in patients with anorexia and bulimia nervosa Eur Arch Psychiatry Clin Neurosci 2000 250 86 92 10.1007/s004060070040 10853924

[B50] Muller K Fach C Diederich F Fluorine in pharmaceuticals: Looking beyond intuition Science 2007 317 1881 1886 10.1126/science.1131943 17901324

[B51] Zhao K Lim DS Funaki T Welch JT Inhibition of dipeptidyl peptidase IV (DPP IV) by 2-(2-amino-1-fluoro-propylidene)-cyclopentanecarbonitrile, a fluoroolefin containing peptidomimetic Bioorg Med Chem 2003 11 207 215 10.1016/S0968-0896(02)00384-X 12470715

